# The Educational Problem—Victory

**Published:** 1884-09

**Authors:** 


### Editorial.

### THE EDUCATIONAL PROBLEM—VICTORY.

If this were a political weekly, instead of a staid scientific and professional journal, the INDEPENDENT PRACTITIONER would probably hoist at the head of this column a rampant rooster, in celebration of the long step in advance made by the Association of Colleges, a report of whose meeting may be found in this number. The accomplishment of the event is one in which this journal takes some pride, for we believe it has been largely instrumental in bringing it about. Our readers may now know for what purpose we have given space to the various articles upon this subject, contributed by both European and American writers.

The Degree of Doctor of Dental Surgery is distinctively an American degree. Dental Colleges exist nowhere, save in America. It was their organization that placed American dentistry ahead of the world. As long as students were required to complete a full curriculum of study, and none but those possessed of the requisite preliminary qualifications were permitted to matriculate, we easily maintained our lead, and foreign students resorted to our schools to finish their professional education. (It should be remembered that graduates of most of the foreign Universities are supposed to be fully qualified to practice either Medicine, Law, Theology, or Dentistry, without special study.) In an evil hour some of our colleges agreed to accept twelve years' practice as the equivalent for one term of schooling. The downhill road once entered upon, further reductions soon followed. The practitioner of *five* years' standing was accepted as a second year student. *Facilis descensus Averni*, and soon the five year practitioner was only required to show himself occasionally at lectures. It was but one step further to allow the practitioner to present himself for graduation upon a mere examination at the close of a term, and this in many cases degenerated into a mere form. There was but one deeper depth in college degradation, and the diplomas of regularly chartered schools were soon almost openly offered for sale. Dentists who had obtained some standing in the profession (and some who had not) were ambitious to affix the dubious title "Professor" to their names, and covetous of the fees that could be earned, and schools multiplied. The competition for students became active, and the standard continued to droop. Some of our most reputable colleges entered upon the work of granting degrees upon examination only, and this soon became almost the rule. It was urged that the possession of the necessary qualifications, however obtained, should be the criterion, forgetting that the diploma of a college is not an indication of the acquisition of a given amount of practical knowledge, but the evidence that the graduate has pursued a specified course of study. A diploma does not make a dentist, nor a physician; but it should be testimony that its possessor has had the opportunity to qualify himself to be one. To their credit be it said, a few of our colleges had not bowed the knee to the golden calf, but had stoutly maintained their integrity, and refused to grant their honors to any, except such as had spent the requisite time within their walls.

Formerly it had been the educated class of foreigners who had sought our schools, and they had come for legitimate training. But now charlatans of all grades came diploma-hunting. Uneducated men, who were even ignorant of the English language, and therefore unable to profit by the lectures, stoutly represented themselves as five-year practitioners, and after a brief sojourn in this country returned to Europe armed with college diplomas, and the colleagues of reputable graduates. It is little wonder that the American degree began to fall into disrepute, and the graduates of European Universities refused to place themselves upon a level with these pretenders and impostors. The character of European students underwent a complete change, and in place of intelligent gentlemen, most of our schools began to be infested with mere adventurers and charlatans. The low grade of professional education in this country was reflected upon our American representatives abroad, and they felt the disgrace keenly. American dentists at home began to vent their dissatisfaction in our societies, and there were ominous mutterings in all directions.

It was at this crisis that the present publishers of the INDEPENDENT PRACTITIONER thought they saw an opportunity to do some good work for their beloved profession. When they purchased this magazine and organized their Association, it was their avowed determination and distinct understanding with each other, that the reorganized journal should begin an agitation for reform in our schools. But it was necessary to proceed cautiously, and to avoid the imputation of wanton iconoclasm. Both in the "Editorial" and "Original Communications" departments, it began immediately to lead up to the subject, and to call the attention of the profession to the educational status. Statistics and facts were gathered, correspondence with representative men unconnected with the schools was carried on, and shortly after the last annual meeting of the stockholders it was decided to speak in yet plainer tones. It was determined that as soon as an opportunity was offered it would lay before the profession all the facts in some case of graduation *sine curriculo*, regardless of whom it affected, and appeal to the profession for judgment upon a practice that was fast becoming universal. One of the publishers was himself the Dean of a Dental College, but it was fully understood that if a case arose implicating his own school there should be no staying of our hands, and to this he cordially agreed.

The instance presented itself, and true to our resolution the charges brought in our June number were published, although they involved the names of some of our best friends. It was not in enmity to them, or in hostility to the Baltimore College, that we gave the article place. There was a principle involved, that we deemed of vital importance to the profession. Those who had not comprehended our purpose had denounced us for articles already published, and we were loudly threatened by others who did not see the end at which we aimed, if we gave to the world specific charges. We submit that in view of these things it required some courage to open the matter in the way in which we did it. But we trusted to a hoped-for victory for our justification. We believed the granting of other than honorary diplomas to even the most reputable dentists, upon a mere examination, was vicious in principle, and demoralizing in its tendency. We were quite as certain that the allowing of five, or even ten years' practice, as the equivalent of one course of lectures, was almost equally wrong, and we thought that when specific cases were presented the profession would not be slow to perceive it. The victory has come sooner than we anticipated, for the colleges themselves have recognized the propriety of the principle, and have not waited for the voice of the profession to be heard. We believe the action taken at New York and Saratoga is fraught with more of good to the educational interests of the profession than anything that has transpired within our knowledge. Our end is gained. The discussion of this subject will now be dropped, so long as the colleges stand by their agreement. We do not think a single reputable school will refuse to come into the arrangement. Certainly, they cannot afford to do so, and should they decline it will be the duty of such State Societies as have proper legislation to sustain them, to refuse to acknowledge their diplomas, and to prosecute every graduate who attempts to practice under them. Colleges can act their pleasure about joining the Association, but they must conform to the standard.

Should any of the schools, after the session of 1884–85, go back to the old standard, the INDEPENDENT PRACTITIONER will publish every well attested instance, and warn the profession against them.

And now, concerning the discussion between the Baltimore Colleges. Notwithstanding our pruning of every article submitted to us, much has been allowed to appear in these pages which we regretted, but which under the circumstances we felt powerless to prevent. Both sides have been given two opportunities for a hearing. With the publication of the article by the Faculty of the Baltimore College in this number, the discussion is closed, so far as this journal is concerned. If the spectacle of this washing of dirty linen in public has been a painful one, we think the end has justified it. Both sides may have the gratification of knowing that they have contributed toward a very desirable consummation. The men whose names have been used can feel assured that their own complaisance, while in a painful position, will be fully appreciated by a grateful profession. Our contributors, foreign as well as home, who have furnished us articles that have assisted in the work, are entitled to the thanks of all who love their calling.

In conclusion, it is but fair for us to state our opinion, that if the Baltimore College has been guilty of any "irregularity" in the granting of degrees, it was one that was far too *regular*, for while it does not deny the conferring of diplomas *sine-curriculo*, it has but followed a practice that has become too general among our schools, and one that it did not originate; a custom that dates back for years, but which in common with other institutions that have pursued the same course, it now definitely agrees to abandon.

The Dental Department of the University of Maryland has, we believe, graduated but two classes, and as it is governed by the University regulations, it has, so far as we know, never granted its honors except in course. Sufficient evidence has been submitted to convince us that, in the preparation and forwarding for publication of the article in our June number, there was no collusion between members of its Faculty and the author.

Dr. Hopkinson has the satisfaction of knowing that his article has produced a most decided sensation, and wrought results that he could scarcely have anticipated. He has, we believe, never been connected officially with the University, and there is no evidence to prove that in writing his article he was prompted by other motives than those of love for his profession. It was the simple duty of every dentist to do what he could to break up a general practice that he believed to be wrong, and Dr. Hopkinson presented the instances with which he was most familiar.

The graduates whose names have been used received their diplomas upon precisely the same grounds that other good men have obtained theirs, and are certainly no more culpable. If they chose to leave the one school, whose honors could not be obtained save by spending more time than they felt they could afford, and go to another having less stringent regulations, it was their legal right so to do.

With any personal differences that may exist between the Faculties of the two institutions, neither our readers nor ourselves have, or desire to have, anything whatever to do.

